# Bone‐strengthening effects and safety of compound peptides from skin of *Chiloscyllium plagiosum* and *Mustelus griseus*

**DOI:** 10.1002/fsn3.1438

**Published:** 2020-02-13

**Authors:** Xin‐Heng Xu, Peng‐Fei Lv, Tong‐Xin Wang, Bao‐Xuan Wang, Yan Shi, Bi‐Xue Wang, Zheng‐Rou Meng, Qing‐Xi Chen, Jiang‐Xing Zhuang, Yule‐Yue Wang

**Affiliations:** ^1^ Key Laboratory of the Ministry of Education for Coastal and Wetland Ecosystems School of Life Science Xiamen University Xiamen China; ^2^ Fujian Provincial Key Laboratory of Neurodegenerative Disease and Aging Research Institute of Neuroscience School of Medicine Xiamen University Xiamen China; ^3^ Tekwon Genetic Technologies Ltd. Xiamen China

**Keywords:** bone trabecular, bone‐strengthening effects, compound peptides, shark skin, subacute toxicity

## Abstract

Fish processing produces a lot of by‐products highly containing large amount of proteins which mainly consist of collagen, implying great potential value for application as nutraceutical ingredients. In present study, two kinds of sharks, *Chiloscyllium plagiosum* and *Mustelus griseus*, were used as raw material to gain three kinds of “compound peptides” (CPs) by enzymolysis, FCP (CPs from the flesh of *C. plagiosum*), SCP (CPs from the skin of *C. plagiosum*), and SMG (CPs from the skin of *M. griseus*). According to a series of constituent analysis, the molecule weights of FCP, SCP, and SMG were under 800 Da; amino acids composition analysis of FCP, SCP, and SMG showed that there were high glycine, proline, and hydroxyproline and low cysteine contents in SCP and SMG, which is the characteristic of collagen peptides; their total protein contents were 87.500%, 91.875%, and 95.625%, respectively; and heavy metal contents of CPs were all beneath national standards. After three kinds of CPs were administrated intragastrically to C57BL/6 mice at a total dosage of 15 g/kg, bone‐strengthening effects of SCP and SMG were manifested by osteoblasts activity promotion, bone mineral density (BMD) increase, and marrow adipocyte number decrease, yet nonsignificant effects were shown in FCP group. No index showed toxicity of SCP and SMG in subacute toxicology trial, indicating their safety as functional foods. Herein, industrial application foundation of the skins from these two sharks was explored but more efforts should subsequently be implemented for further exploitation.

## Graphical abstract

Our research showed that the skin of two kinds of sharks Chiloscyllium plagiosum and Mustelus griseus might have a novel application as extractive raw materials of functional food—a hydrolysate which we named compound peptides. They are easy to produce and possess bone‐strengthening effects and nontoxic, laying a foundation for industrial production.

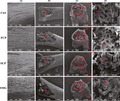

## 1 INTRODUCTION

Over the past several decades, interest has expanded rapidly in the utilization of marine protein supplements. Marine source proteins and peptides, in terms of their structures and amino acid sequences, exert a various of biological activities including antimicrobial, antioxidant, anticancer, antihypertensive, anticoagulant, immunomodulatory, and antidiabetic effects (Cheung, Ng, & Wong, 2015; Wang et al., 2014). Terrestrial animals such as pig and cow have some zoonotic diseases, making their protein dangerous for human, and some regions have religious restrictions to these protein resources (Kittiphattanabawon, Benjakul, Visessanguan, Nagai, & Tanaka, 2005). Aquatic animals are rich in collagen with a wide range of sources, low price, easy processing, and high biosafety for no zoonosis, which contributes to the value of marine protein resources (Zhang et al., 2007).

Fish processing produces a lot of by‐products, such as skin, bone, scales, and fins, which not only causes great wastes, but also results in environmental pollution. Thus, to avoid these issues, fish by‐products need to be exploited. It has been revealed that these wastes are rich in protein resources, where protein content can reach 80% of dry mass, including functional peptides (Najafian & Babji, 2012; Rawat, Joshi, Joshi, & Atheaya, 2006; Veeruraj, Arumugam, Ajithkumar, & Balasubramanian, 2015). This indicates the great potential of fish by‐products for utilization.

*Chiloscyllium plagiosum*, a kind of small sharks inhabiting onto the water bottom, are distributed in the Indo‐Western Pacific region. They are sluggish and tender, generally live in the coastal and rocky seabed, and used as one of the most popular cartilaginous fish for food. The liver of *C. plagiosum* makes up 75% of the weight of viscera and possesses bioactive components (Zhang et al., 2013). *Mustelus griseus* is classified into *Chondrichthyes Carcharhiniformes Triakidae Mustelus*, mainly distributed in the Northwestern Pacific. In previous research, there were five specific peptides isolated from *M. griseus*, which showed strong reactive oxygen species (ROS) scavenging activities ( Wang et al., 2014). At present, the utilization of these two sharks is still mainly at the scope of industrial leather production and food processing.

Bone is a complex organism composed of bone matrix and bone minerals. Collagen accounts for over 90% of bone matrix, and the remains are noncollagen protein (Viguet‐Carrin, Garnero, & Delmas, 2006). In addition, bone is a highly differentiated and bioactive tissue, and the metabolism of bone tissue is a dynamic equilibrium process, which implicates not only the formation of bone tissue but also the absorption of bone tissue (Pierrefite‐Carle, Santucci‐Darmanin, Breuil, Camuzard, & Carle, 2015). Calcium in bones is deposited by hydroxyapatite and fixed by bone collagen. Therefore, it is beneficial to bone metabolism that the amino acids or oligopeptides necessary for the synthesis of collagen are supplemented (Moskowitz, 2000). Recent clinical study has shown that aquatic collagen peptides significantly relieved pain and bone function in osteoarthritis patients (Bello & Oesser, 2006). And alleviation of osteoarthritis pain in the knee or hip was noted in a research of patients treated with 10 g collagen hydrolysate daily in 2‐month duration (Adam, 1991). But studies referring to bone‐related efficacy of shark source peptides have not been reported.

In this study, the skins of *C. plagiosum* and *M. griseus* were used as raw materials to gain two kinds of compound peptides (CPs) by enzymolysis, SCP (CPs from the skin of *C. plagiosum*), and SMG (CPs from the skin of *M. griseus*). Meanwhile, FCP (CPs from the flesh of *C. plagiosum*) were prepared as a contrast sample. The three kinds of CPs’ components were measured, their effects on C57BL/6 mice bones were studied, and their toxicity to the mice was determined, so as to lay an experimental foundation for improving shark skin utilization rate and alleviating by‐product pollution issues.

## 2 MATERIALS AND METHODS

### 2.1 Materials and chemicals

*Chiloscyllium plagiosum* and *Mustelus griseus* were derived from Xiamen Tekwon Genetic Technologies Ltd. and frozen at −20℃ for preservation. Papain (2,000 U/mg), Hematoxylin Staining Solution, and Eosin Staining Solution were purchased from Sangon Biotech Co., Ltd., Shanghai, China. Optimal cutting temperature (O.C.T) was obtained from Sakura Finetek, Inc., Torrance, USA. Pentobarbital sodium was from Sigma‐Aldrich, USA. All other reagents utilized in this research were of analytical grade.

### 2.2 Animal and diet

A total of 140 C57BL/6 mice were obtained from Xiamen University Laboratory Animal Center, among which 100 mice were female and 40 were male. They were divided into two groups, 60 female mice for bone‐strengthening effects examine and 80 mice (40 female and 40 male) for subacute toxicity assay. They had been evaluated to ensure the general health status and acceptability for study purposes for 2 weeks before the research. Male mice weighted about 21 ± 0.5 g, and Female mice were around 17 ± 0.5 g. The animals were housed at gender‐apart cages with free access to standard pellet diet and water and kept on 12 hr‐light/12 hr‐dark cycle under the condition of 21–22℃ temperature and 60%–65% humidity.

### 2.3 Preparation of CPs

Three kinds of compound peptide powders, FCP (CPs from the flesh of *C. plagiosum*), SCP (CPs from the skin of *C. plagiosum*), and SMG (CPs from the skin of *M. griseus*), were extracted from two kinds of sharks, *Chiloscyllium plagiosum* and *Mustelus griseus*, using same process. The body of two sharks was defrosted under room temperature (RT) tap water and cleaned with ultrapure water before cutting the tissues into about 1× 1 cm small pieces, and then adding 1L 10% isopropanol aqueous solution to 100 g of each tissue, and then put them at 4℃ for 12 hr. After that, 10% isopropanol aqueous solution was renewed and left for another 12 hr before it was rinsed with ultrapure water at RT until odorless. The tissues were heated in 1 L ultrapure water, with continuous stirring for 30 min after boiled. After cooling, the tissues and supernatants were homogenized at 2,000 r/min for 3 min to form homogenates. Then, the pH was adjusted to 5.0 with HCl and the volume was fixed to 1 L using ultrapure water. For peptides extraction, the homogenates were added 1 × 10^7^ U (5 g) papain to hydrolyze in 50℃ for 4 hr before boiling for 30 min to stop hydrolysis. To remove sediments and obtain peptide powders, the homogenates were centrifuged for 25 min at 21,612 × *g* and the supernatants were freeze‐dried to derive the powder.

### 2.4 MALDI‐TOF‐MS analysis

The analysis was implemented by Xiamen Medical College. In short, the CPs’ samples needed to be desalinated by a C18 column (Acquity UPLC Symmetry C18; 5 μm; 180 μm × 20 mm) with a flow rate of 0.3 μl/min before loading. One microliter of CPs solution (2 mg/ml) and 1 μL α‐cyanocinnamic acid were mixed and spotted onto the sample target plate. After the mixture solution was volatilized to crystallization, the samples were analyzed by MALDI‐TOF‐MS. The mass examination of MALDI‐TOF‐MS condition was mass range 150–2,250 *m/z*, nitrogen laser 337 nm, acceleration voltage 25 kV, detector voltage 1.8 kV, and analyzer vacuum degree 1 × 10^–8^ Torr.

### 2.5 Amino acid composition and total protein content

Amino acid composition was carried out in accordance with the guideline of national standard “national food safety standard—determination of amino acids in food” (GB 5009.124–2016) by Fujian Inspection and Research Institution for Product Quality (Fuzhou, China). The amino acids were as follows: aspartate (Asn), threonine (Thr), serine (Ser), glutamate (Glu), proline (Pro), glycine (Gly), alanine (Ala), valine (Val), cysteine (Cys), methionine (Met), leucine (Leu), isoleucine (Ile), tyrosine (Tyr), phenylalanine (Phe), histidine (His), lysine (Lys), arginine (Arg), and hydroxyproline (Hyp). Briefly, CPs were hydrolyzed into free amino acid by 6 M hydrochloric acid at 110 ℃ for 6 hr. After separation by ion‐exchange column (sulfonic cation exchange resin), color reaction was generated with ninhydrin solution, and then, the amino acid content was determined by visible light spectrophotometer at wavelength 570 and 440 nm. Among 17 amino acids, hydroxyproline was determined by Hydroxyproline Assay Kit (Solarbio Co., Ltd., China). The samples were hydrolyzed by HCl (same conditions as mentioned above) to released Hyp, which was then oxidized by chloramine T to produce an oxide containing pyrrole rings. The oxides reacted with p‐dimethylaminobenzaldehyde to form a red compound which could be quantitative at wavelength of 560 nm via Hyp standard curve.

Total protein content detection was implemented in accordance with the guideline of national standard “national food safety standards—determination of protein in food” (GB 5009.5–2016). Briefly, CPs were digested under catalytic heating, and the resulting ammonia was connected to sulfuric acid to form ammonium sulfate. Then, ammonia was freed and gasified with alkalization distillation, absorbed with boric acid, and titrated by hydrochloric acid standard solution. Total protein content was calculated according to the consumption of acid by formula below:

$$X=\frac{(V1-V2)\times c\times 0.014\times 100}{m\times V3}\times k\times 100$$

*V1*: consumed volume of hydrochloric acid by the sample, mL; *V2*: consumed volume of hydrochloric acid by blank control, ml; *c*: concentration of hydrochloric acid, M *m*: mass of the sample, g; *V3*: absorption volume of the digestive liquid, ml; *k*: conversion factor of nitrogen to protein, 5.79 for collagen or high collagen tissue, 6.25 for common and low collagen tissue; and X: mass of total protein in the sample, g/100 g.

### 2.6 Element content analysis

The analysis was performed in compliance with the procedure described in “national food safety standards—determination of multi‐elements in food” (GB 5009.268–2016) by Fujian Inspection and Research Institution for Product Quality, using Inductively Coupled Plasma Mass Spectrometry (ICP‐MS). Briefly, after the sample was dissolved, it was determined by ICP‐MS with external standard method. It is qualitative in terms of the specific mass number (mass to charge ratio, *m/z*) of the element. The strength ratio of the quality spectrum signal with internal standard signal was directly proportional to the concentration of the element, which was utilized for quantitative measurement of samples. The instrument condition: radio frequency power 1,500 W, plasma gas flow rate 15 L/min, carrier gas flow rate 0.80 L/min, sampling depth 8–10 mm, auxiliary gas flow rate 0.40 L/min, helium gas flow rate 4–5 ml/min, and atomization chamber temperature 2℃.

### 2.7 The effect of CPs on mice bone

Sixty female mice were randomly divided into 4 groups, 15 mice in each, and labeled as control group, FCP group, SCP group, and SMG group. The experimental groups were administered intragastrically 50 mg/ml CPs of each kind, respectively, while control group was administered intragastrically distilled water. Intragastric administration was performed at a volume of 20 ml/kg body weight (BW) each time every other day, and the treatment lasted for 30 days (15 times) to a total dose of 15 g/kg (CPs: BW).

After treatment, the mice were sacrificed by cervical dislocation; then, we took femurs and tibiae of mice, removed flesh and muscular tissue on the bone surface, and fixed bones with 4% paraformaldehyde (PFA) for at least 24 hr. We took femurs for weight detection and rinsed tibiae samples with ddH_2_O twice followed by drying to constant weight at the temperature of 50℃.

#### 2.7.1 Scanning electron microscopy (SEM) of mice tibiae

We used SEM to detect three parts of tibia, the surface of middle part of tibia shaft, the cancellous substance in proximal tibia metaphysis (PTM), and the cross section of PTM at osteoepiphysis side**.** The dried tibiae samples were incised to expose the cancellous bone and pasted mildly on the electroconductive adhesive tape in a given orientation. Then, they were placed inside the sputter to vacuumize and spray platinum for about 1 min. Afterward, the sputtered samples were observed with scanning electron microscope (JSM‐6390LV, JEOL, Japan) at 30×, 100×, and 150 × magnification at a voltage of 10 kV.

#### 2.7.2 Hematoxylin and Eosin (HE) staining of mice tibiae

The tibiae samples were decalcified with EDTA decalcified solution (BBI Life Sciences Co., Ltd., Shanghai, China) for at least 2 days until the bones were pliable rather than tough and fragile. After decalcification, tibiae samples were rinsed with ddH_2_O and soaked in 50% ethanol before dehydration procedure. Automatic tissue hydroextractor (Leica TP1020, USA) was applied for dehydration with program: 70% ethanol for 2 hr, 80% ethanol for 1 hr, 95% ethanol for 30 min twice, 100% ethanol for 30 min twice, ethanol–xylene mixture for 15 min, xylene for 10 min twice, and paraffin for 45 min twice. Then, paraffin embedding, sectioning, HE staining, and histological examination were executed.

### 2.8 Subacute toxicology of CPs

Eighty mice, forty for each sex, were divided into 4 groups, ten males and ten females for each group. The labeling and administration method were introduced above. After treatment, the body weight (BW) of mice was weighted. Then, they were anaesthetized by pentobarbital sodium, fixed on operating table, and implemented heart perfusion for replacement of blood to PBS, and then injected 4% PFA to replace PBS before taking heart, liver, spleen, lung, and kidney for macroscopic morphology, viscera coefficient (viscera: BW), and histopathologic examination with HE staining.

In brief, for HE staining, the viscera samples were dehydrated using 30% saccharose until the tissues sank to the bottom; afterward, O.C.T embedded, frozen at −80℃, sliced with frozen section machine (CM1950; Leica, USA), and HE‐stained for morphology observation.

### 2.9 Statistical analysis

Independent samples were analyzed by *t* test (*p* < .05). The intergroup comparison analysis was performed using one‐way analysis of variance (ANOVA), (*p* < .05). GraphPad Prism (version 6.0.1) and SPSS (version 22.0) were used for graph construction and statistical analysis.

## 3 RESULTS

### 3.1 The molecular weights of CPs

**Figure 1:**
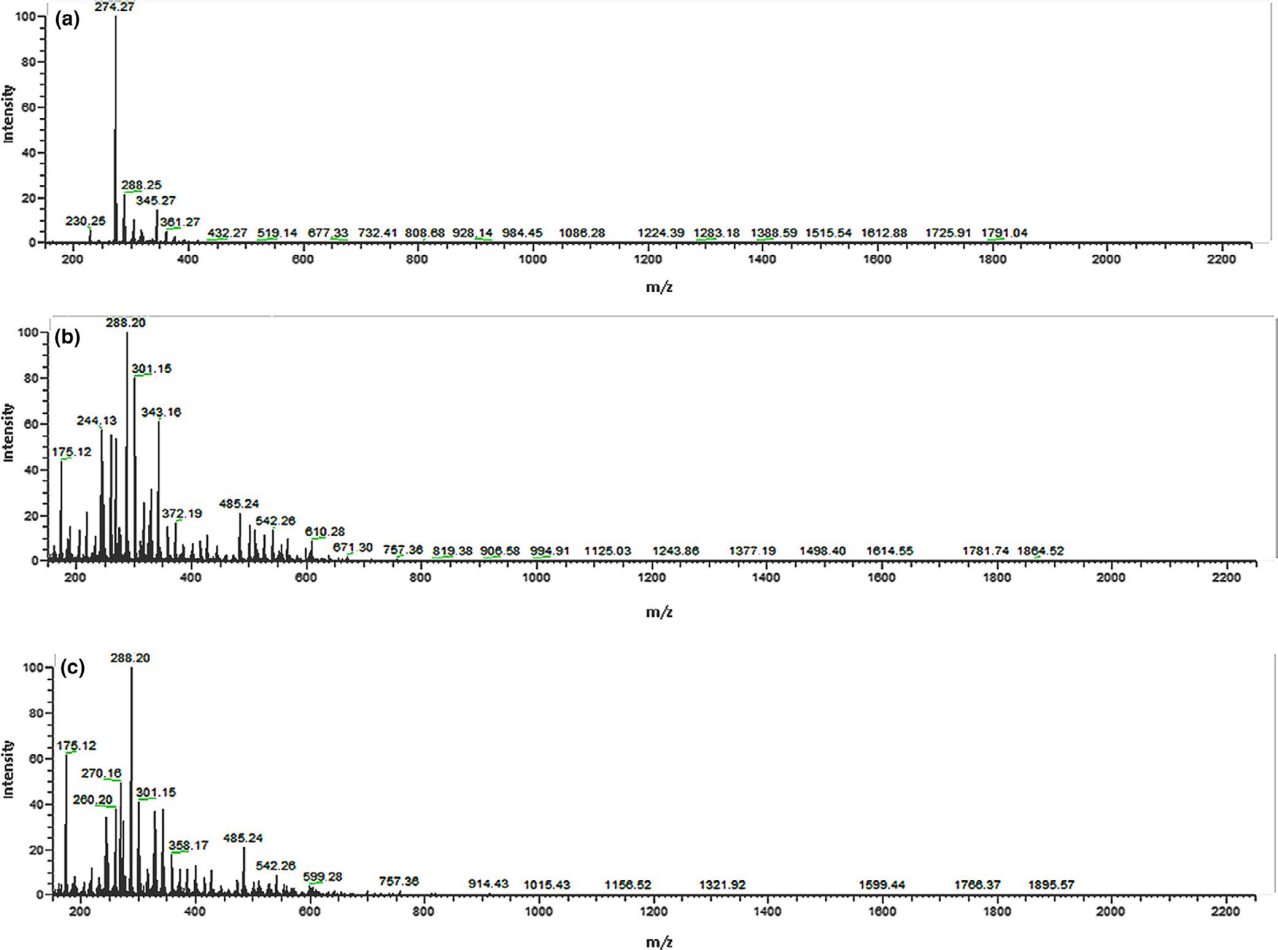
MS spectra of FCP (A), SCP (B), and SMG (C) by MALDI‐TOF‐MS

The molecular weights of three kinds of CPs, FCP, SCP, and SMG, were evaluated by MALDI‐TOF‐MS (Figure 1). The results showed that three kinds of CPs were composed of a variety of ingredients possessing different molecular weights. What was interesting was that the composition of SCP and SMG was similar while not that of FCP. This discovers provided primary explanation of bio‐efficacy similarity of these two kinds of shark's skin from component. It was uncovered that molecular weights of these ingredients were lower than 800 Da, demonstrating that these CPs consisted of low molecular substances and oligopeptides.

### 3.2 Amino acid composition and total protein content

The amino acid composition and total protein content of three kind of CPs are listed in Table 1. As table shows, SCP and SMG have similar amino acids proportion, and both contain relatively higher contents of glycine, alanine, proline, and hydroxyproline, while FCP contain relatively higher contents of glutamate, leucine, phenylalanine, lysine, and cysteine. The skins from two kinds of sharks have more total protein contents than the flesh of *C. plagiosum.* The total protein contents of FCP, SCP, and SMG were 87.500%, 91.875%, and 95.625%, respectively.

**Table 1:** Amino acid components and total protein contents of FCP, SCP, and SMG

Amino acid	FCP (g/kg)	SCP (g/kg)	SMG (g/kg)
Asn	77.7 ± 1.8^a^	49.4 ± 1.7^b^	56.8 ± 2.4^c^
Thr	47.0 ± 3.4^a^	29.4 ± 1.8^b^	32.7 ± 1.9^b^
Ser	37.3 ± 0.9^a^	32.4 ± 2.0^b^	29.8 ± 2.2^b^
Glu	113.8 ± 3.2^a^	77.3 ± 2.1^b^	79.8 ± 1.6^b^
Gly	47.5 ± 0.9^a^	160.5 ± 2.5^b^	135.2 ± 1.1^c^
Ala	58.0 ± 1.6^a^	109.5 ± 2.0^b^	97.8 ± 2.4^c^
Cys	7.9 ± 0.3^a^	4.0 ± 0.4^b^	3.7 ± 0.2^b^
Val	27.7 ± 1.8^a^	21.8 ± 0.3^b^	23.0 ± 1.6^b^
Met	24.1 ± 1.9^a^	15.8 ± 0.3^b^	16.9 ± 1.2^b^
Ile	33.5 ± 1.1^a^	21.6 ± 0.9^b^	25.8 ± 1.1^c^
Leu	61.9 ± 1.3^a^	31.0 ± 0.7^b^	39.5 ± 1.8^c^
Tyr	44.7 ± 1.9^a^	22.3 ± 1.9^b^	25.8 ± 2.1^b^
Phe	35.7 ± 1.4^a^	17.1 ± 1.2^b^	18.0 ± 0.9^b^
Lys	70.1 ± 1.7^a^	35.1 ± 0.8^b^	43.7 ± 1.1^c^
His	22.8 ± 0.2^a^	12.0 ± 0.3^b^	12.7 ± 0.6^b^
Arg	47.1 ± 0.8^a^	52.4 ± 1.2^b^	60.4 ± 0.4^c^
Pro	58.2 ± 0.7^a^	86.0 ± 1.1^b^	85.3 ± 1.3^b^
Hyp	1.3 ± 0.1 ^a^	30.0 ± 0.4 ^b^	43.1 ± 5.0^c^
Total protein	875.00 ± 5.8^a^	918.75 ± 4.1^b^	956.25 ± 3.2^c^

Note

Different letters in the same row indicated significant differences (*p* < .05).

### 3.3 Heavy metal and macroelement content

The result of heavy metal contents is shown in Table 2. The contents of arsenic, mercury, and lead are corresponded to the safety quantity according to “national food safety standards—health care food” (GB 16740–2014). Magnesium (Mg), potassium (K), sodium (Na), calcium (Ca), ion (Fe), zinc (Zn), selenium (Se), and copper (Cu) were detected and listed in Table 3. As the table showed, Ca is more in SCP and SMG than in FCP, which was beneficial for bone strengthening.

**Table 2:** Heavy metal contents of FCP, SCP, and SMG. The maximum limit indexes referring to “national food safety standards—health care food” are listed in the rightmost column

Elements	FCP (mg/kg)	SCP (mg/kg)	SMG (mg/kg)	Limitation (mg/kg)
Arsenic	0.32 ± 0.01^a^	0.16 ± 0.02^b^	0.22 ± 0.01^c^	1.00
Mercury	0.23 ± 0.01^a^	0.07 ± 0.01^b^	0.09 ± 0.00^c^	0.30
Lead	0.31 ± 0.02^a^	0.06 ± 0.00^b^	0.22 ± 0.01^c^	2.00

Note

Different letters in the same row indicated significant differences (*p* < .05).

**Table 3:** Macroelement detected in FCP, SCP, and SMG

Elements	FCP (mg/kg)	SCP (mg/kg)	SMG (mg/kg)
Mg	1.23 × 10^3^ ± 4.21^a^	2.36 × 10^3^ ± 3.27^b^	612 ± 2.77^c^
K	3.16 × 10^3^ ± 3.13^a^	1.59 × 10^3^ ± 5.30^b^	293 ± 2.22^c^
Na	4.18 × 10^3^ ± 2.81^a^	7.88 × 10^3^ ± 1.25^b^	2.70 × 10^3^ ± 0.91^c^
Ca	804 ± 1.43^a^	3.31 × 10^3^ ± 2.16^b^	1.17 × 10^3^ ± 1.25^c^
Fe	3.60 ± 0.21^a^	15.51 ± 0.3^b^	7.42 ± 0.13^c^
Zn	15.89 ± 1.22^a^	8.45 ± 0.4^b^	15.03 ± 0.22^c^
Se	2.0 ± 0.01^a^	1.5 ± 0.12^b^	1.2 ± 0.02^c^
Cu	0.46 ± 0.00^a^	0.83 ± 0.01^b^	2.2 ± 0.01^c^

Note

Different letters in the same row indicated significant differences (*p* < .05).

### 3.4 The effect of CPS on mice bone

**Figure 2:**
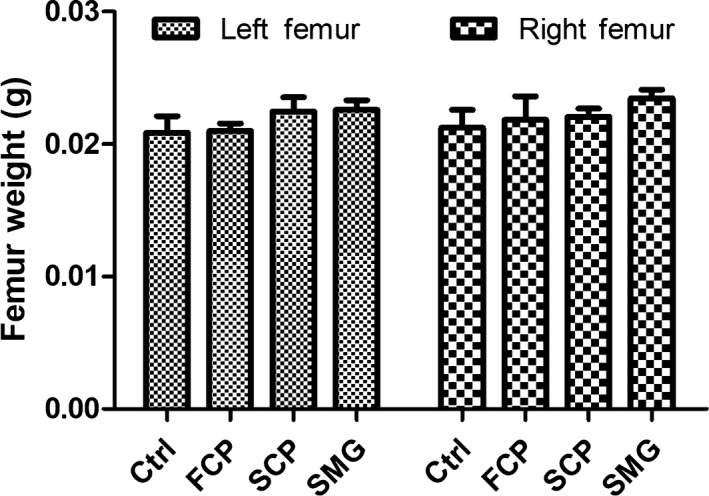
The effect of three kinds of CPs on femur weights of mice

After 30 days’ treatment, the femurs and tibiae of mice were collected. After the flesh and muscle attaching to the bone were stripped, femurs were weighted and tibiae were processed before observation under scanning electron microscopy. The tibiae samples exhibited in figures were chosen randomly. According to the result represented in Figure 2, the increasing of average femur weight was detected in the treatment groups, among which SMG had the highest value while FCP had lowest, but the result of *t* test analysis did not show significant differences (*p* > .05).

**Figure 3:**
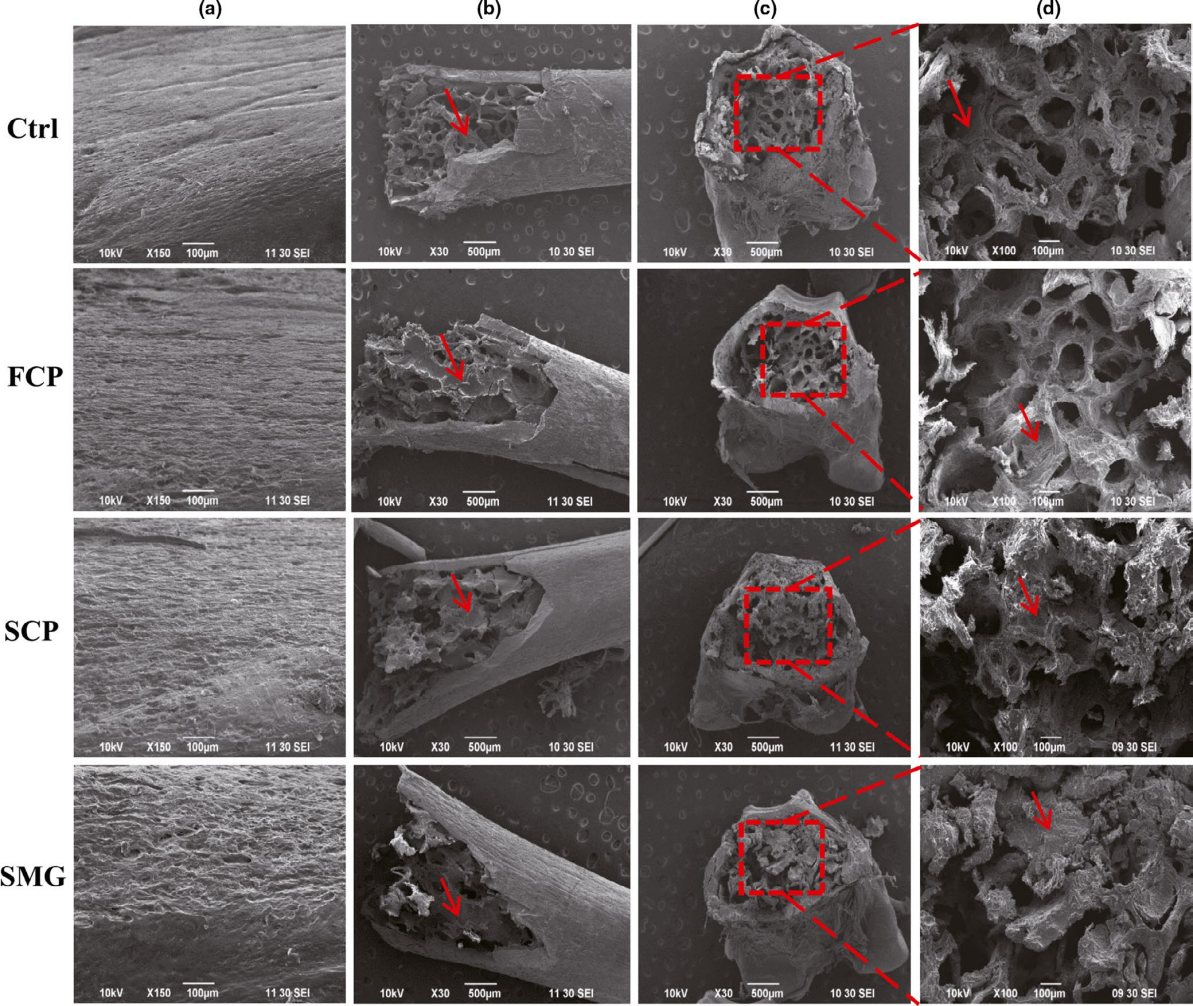
Effects of FCP, SCP, and SMG on bone strengthening detected by scan electron microscope. (a): the surface of middle part of tibia shaft; (b): the cancellous substance in PTM; (c): the cross section of PTM; and (d): magnified image of corresponding red dashed boxes in c. The bone marrows are marked by red arrow

According to Figure 3, the surface of middle part of tibia shaft was relatively smooth, and the arrangement of its collagen fiber bundles was close and consistent compared with the control group, indicating that the bone metabolism was stable and its augmentation tended to stop. However, the bone surfaces of SCP and SMG groups were uneven, rough, and honeycombed, which manifested active bone metabolism, indicating that the osteoblasts on the periosteum have strong activity to form bone matrix, while FCP group presented nonsignificant effect. After drying treatment, the bone marrow was dehydrated and shrunk to form flakes or attach to bone tissues such as bone trabeculae. There were relatively fewer flaky bone marrow tissues in the control group, and most of bone trabeculae was observed clear reticular structure as fewer bone marrow tissues attached to them. The bone marrow abundance was obviously increased in the three experimental groups, presenting flaky, covering the structure of bone trabeculae and filling the interval of cancellous substance, especially in SCP and SMG groups. The results showed that FCP, SCP, and SMG enhanced bone metabolism and promoted marrow tissue abundance with varying degrees, among which FCP had slight effects.

**Figure 4:**
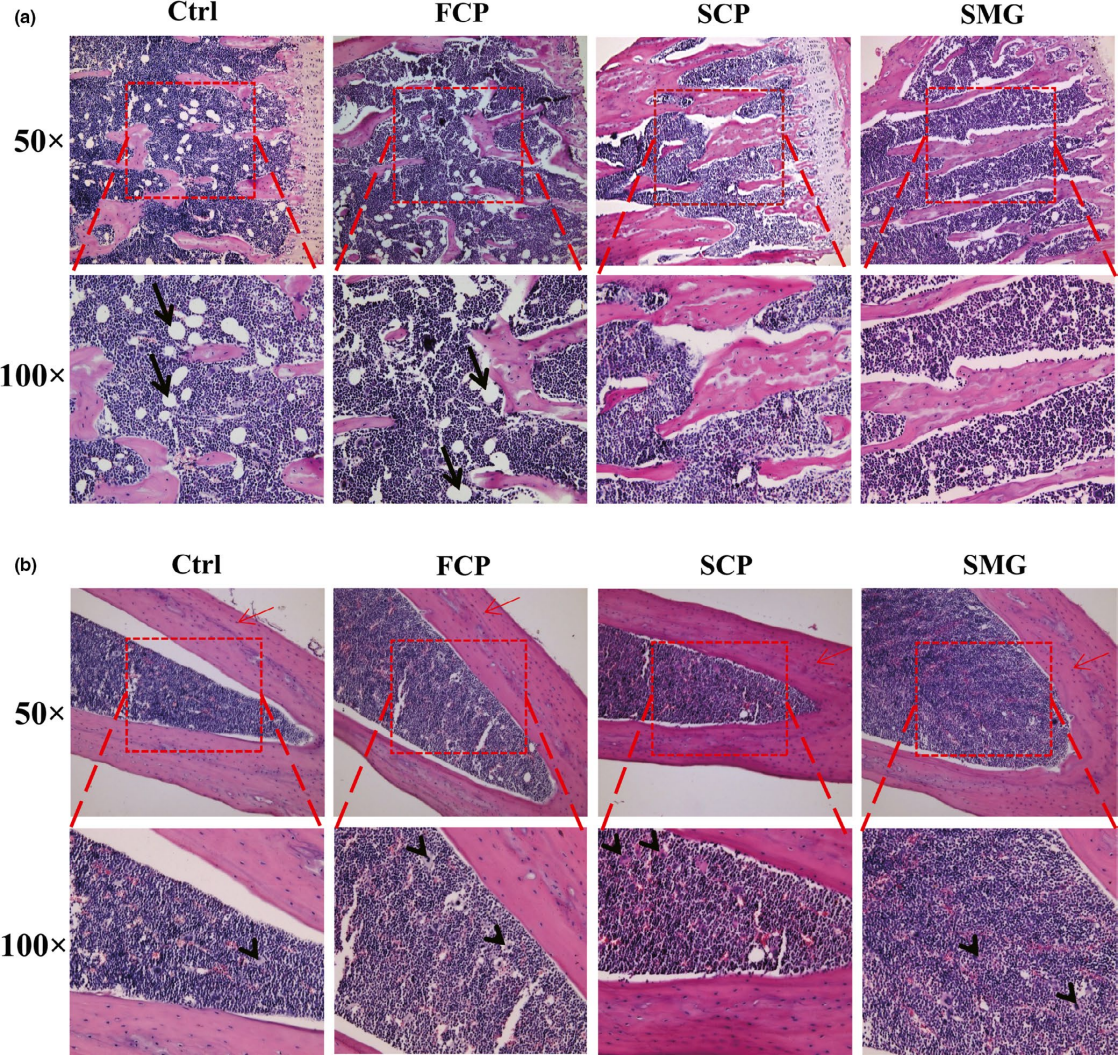
Bone‐strengthening effects of FCP, SCP, and SMG in HE staining assay. (a): sections from PTM. (b): sections from middle tibia shaft. Pictures were taken at two kinds of magnification, 50 × and 100×. The 100 × pictures were obtained by magnifying the image in red dashed frames of corresponding 50 × pictures. Osteocytes are marked by red arrow. Adipocytes are marked by black arrow. Megakaryocytes are marked by black arrowhead

After collecting the tibiae of mice, we made paraffin sections choosing PTM and middle part of tibia shaft. Slices from different groups must be ensured at the same position. The HE staining results are shown in Figure 4. The bone trabeculae tissues in SCP and SMG groups were more abundant and thicker than that in control and FCP groups, revealing that the bones from experimental groups had higher bone density and greater mechanical resistance. And adipocytes, which looked like blank bubble (Figure 4a), were fewer in SCP and SMG groups than in control and FCP groups. That meant the hematopoietic function of SCP and SMG groups was more capable than that of control and FCP groups. The number of osteocytes from middle part of tibia shaft in control and FCP groups were obviously fewer than in SCP and SMG groups (Figure 4b), which showed that the bone generating capability in SCP and SMG groups was much stronger than in control and FCP groups. An interesting discover was that, as shown in Figure 4b, the number of megakaryocyte increased in experimental groups compared with control group, which might imply that the thrombocytopoiesis and clotting function were induced stronger by CPs.

### 3.5 Toxicity analysis of CPs

**Figure 5:**
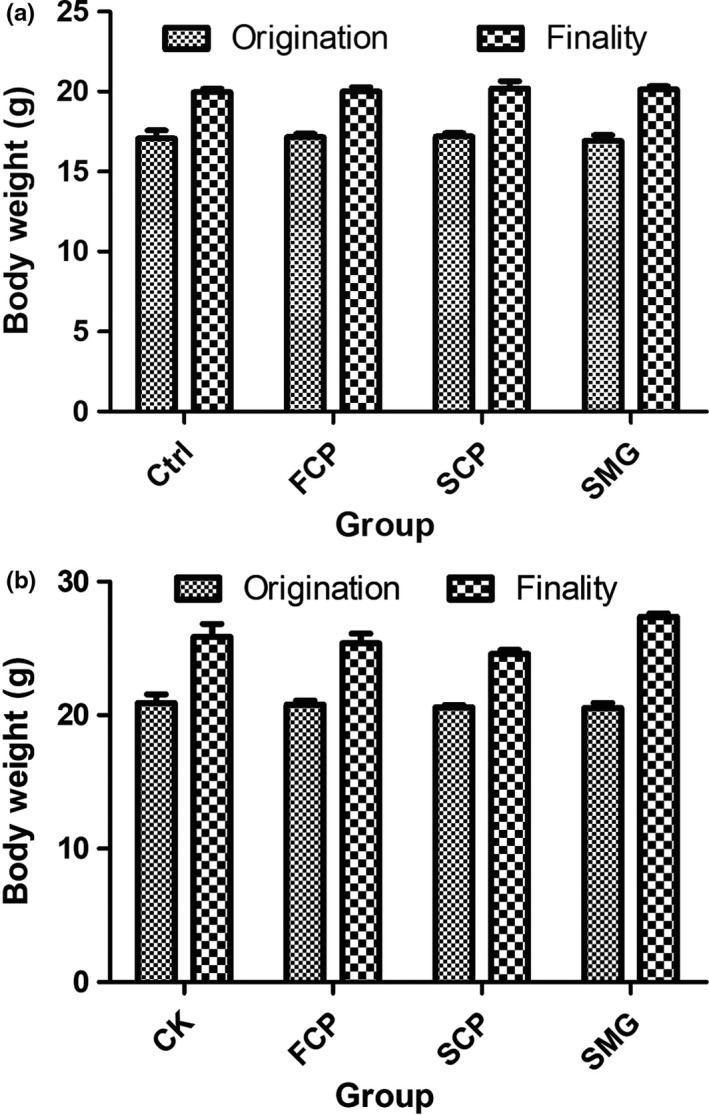
CPs possess no inhibition to mice BW increase. (a): female; (b): male

During the treatment, no mice died. All of mice had healthy and bright fur, and none of them showed skin damage. Behaviors of mice were normal, in which they had common eating, defecating, sleeping, and corresponding responses to external stresses. The growth of mice was all coincident with the normal development process of mouse. Their body weights were shown in Figure 5. The BW of three experimental groups exhibited no significant differences to control group (*p* < .05).

**Figure 6:**
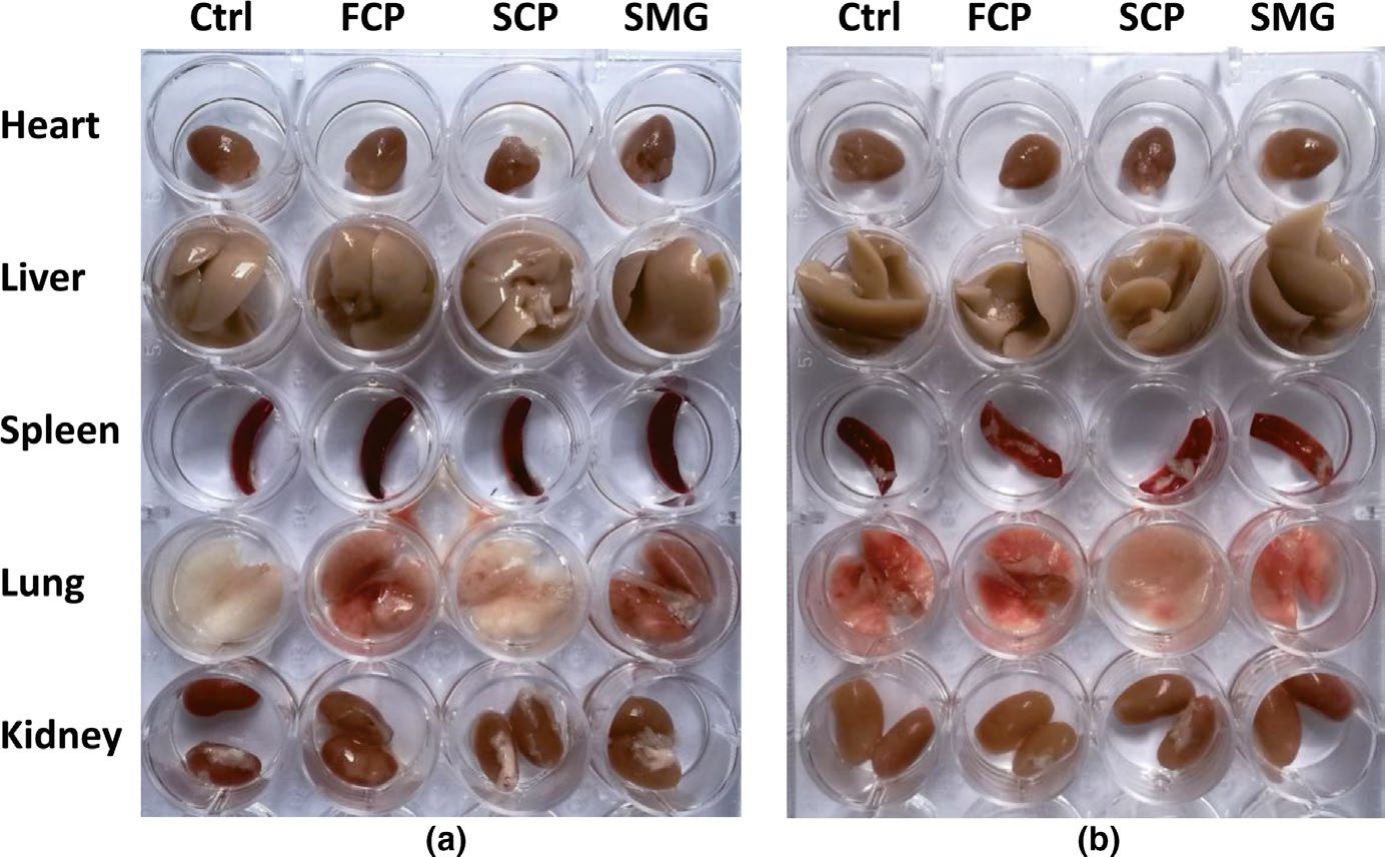
Toxicity evaluation of CPs by macromorphology detection of mice viscera after experiment. Note: Some viscera have blood stasis resulting in their redder color than others. (a): female. (b): male

**Figure 7:**
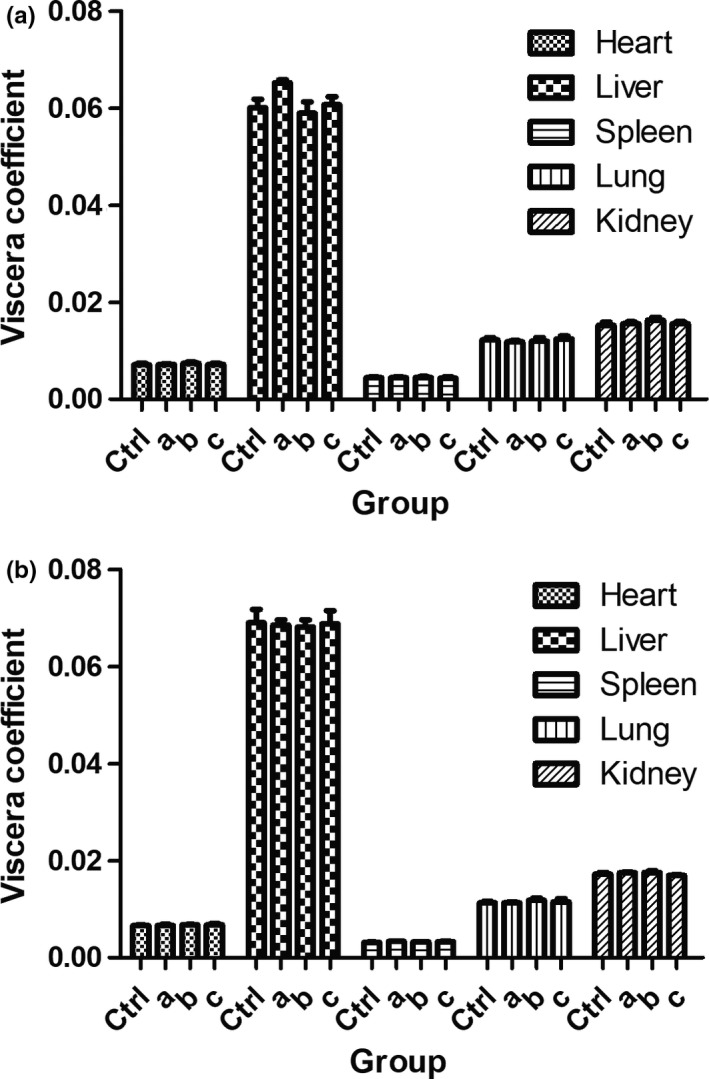
Toxicity evaluation of CPs by viscera coefficient determination. (a): female; (b): male. (a), (b), and (c) represent FCP, SCP, and SMG, respectively

According to the viscera samples from mice, no significant toxicological feature was observed on the morphology of viscera from different groups (Figure 6). There was no hole, damage or abnormality in viscera and they had normal colors and sizes. Viscera coefficient was shown in Figure 7, which indicated no significant differences among groups (*p* < .05).

**Figure 8:**
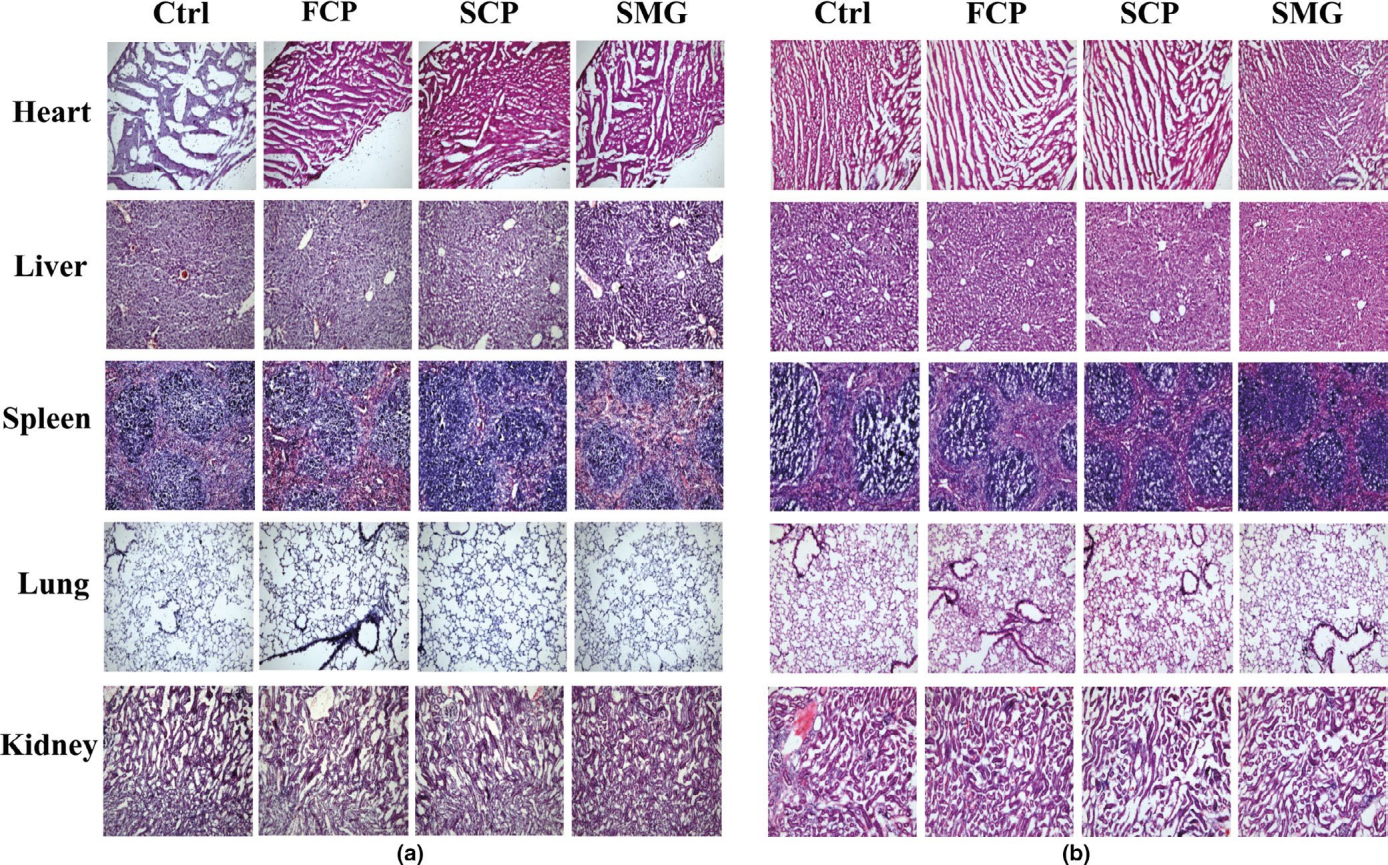
Toxicity of CPs detected by HE staining assay. (a): female mice; (b): male mice. The slices of heart, liver, spleen, lung, and kidney of dose group are shown nonsignificant differentiation to that of control group

HE staining of heart, liver, spleen, lung, and kidney showed that after feeding CPs, there were no toxicological damages within the viscera, of which microscopic structure was complete and clearly layered, and cells were well arranged (Figure 8).

## 4 DISCUSSION

It has been proved that some peptides, which are presented in the inactive form within the protein chains, are activated after their hydrolysis using enzymes (Ngo, Vo, Ngo, Wijesekara, & Kim, 2012; Senevirathne & Kim, 2012; Venugopal, 2011). Hence, potential broad spectrum of bioactivities is contained in the marine fish waste‐derived peptides which have presently gained tremendous interest in functional food and cosmeceutical industries (Pangestuti & Kim, 2017). A considerable amount of literature has been published on collagen and collagen peptides from fish processing wastes, laying a technical foundation for improving the utilization of fish protein resources (Nagai, Araki, & Suzuki, 2002; Nagai & Suzuki, 2000; Xu, Han, & Li, 2010; Yata, Yoshida, Fujisawa, Mizuta, & Yoshinaka, 2001). A research has revealed that fish collagen peptides can enhance the function of endogenous collagen synthesis by post‐transcriptional modification and mineralization (Yamada et al., 2013).

Marine fish proteins have been widely utilized in pharmaceuticals, nutraceuticals, and food applications (Venkatesan, Anil, Kim, & Shim, 2017). The skin of fish contains a high content of collagen, which can reach up to 30% of dry mass (Sun, Hou, Li, & Zhang, 2017). Thus, it is often used as ingredient for collagen and collagen peptides extraction in biological studies (Jeong, Venkatesan, & Kim, 2013; Senaratne, Park, & Kim, 2006; Sionkowska, Kozlowska, Skorupska, & Michalska, 2015). A classical protein sequence of collagen is the cyclic arrangement of Gly‐X‐Y within its helical domain, in which X and Y positions are often proline and hydroxyproline, and they are the most abundant content among various amino acids accounting for about 25%. However, collagen has no tryptophan and seldom cysteine (Moskowitz, 2000). In present study, we extracted flesh and skin peptides and compared their amino acid composition. The result showed higher contents of glycine, proline, and hydroxyproline but lower content of cysteine in the skin than that in the flesh, presenting the characteristic feature of collagen peptides; hence, there should be more collagen content in sharks’ skin than in their flesh.

From our study, the three kinds of CPs have fairly high protein contents reaching up to more than 85%, and the highest of them, SMG, reaches up to 95.625%. The marine hydrolysates reported previously contained total protein content at a range of 70% to 98%, at which the hydrolysate could apply as essential sources of protein (Lassoued et al., 2015; Pei et al., 2010; Wang et al., 2013; Xu et al., 2010). Furthermore, our extractive procedure is efficient, and the CPs we obtained possess high purity on peptides, as well as efficient human ingestion due to a compact molecule weight for intestine absorption, which is beneath 800 Da by MALDI‐TOF‐MS. Various elements and minerals were kept in the procedure in order to remain the inherent nutrient and bioactivity as far as possible, among which calcium was conducive to bone mineralization.

The main anatomical and functional unit of cancellous bone is the trabeculae, which is formed by osteoblasts covered in endosteum. The trabeculae have an irregular network of space in which bone marrow and hematopoietic stem cells produce platelets, red and white blood cells (Young, Lowe, Stevens, Heath, & Deakin, 2006). Osteoblasts play a major role in bone formation, forming osteoid on the periphery which mineralizes to become bone. In osteogenesis, the surface of bone becomes uneven and honeycombed due to the active secretory of osteoblasts and incomplete mineralization of osteoid. In our research, the surface of tibiae became honeycombed and uneven after treated with SCP and SMG at a total dosage of 15 g/kg, while the control group did not and FCP group manifested nonsignificant effect. The result indicated that the osteoblasts were intensified in osteogenesis by the drugs. We speculated the main mechanism was that the peptides were ingested to osteoblasts by endocytosis and utilized directly as ingredients for collagen genesis, and as the very peptides containing high collagen characteristic amino acids, the collagen genesis was facilitated, resulting in osteogenesis acceleration. Hence, it makes sense that SCP and SMG showed high capacity in bone‐strengthening effects, but FCP did not, because they have much higher contents of collagen characteristic peptides than the latter one.

After osteogenesis, osteoblasts will differentiate into osteocytes and remain in the bone lacunae, losing the ability to form bone matrix (Young et al., 2006). The intensity of osteogenesis positively correlates to the quantity of osteocytes in the cortical bone and trabeculae, and the increase of bone mineral density (BMD) can be reflected by the increase of crosslinking degree of trabeculae and area of bone (Simonet et al., 1997). We observed the increase on the area of trabeculae and the quantity of osteocytes after the participation of SCP and SMG. Previous research drew similar conclusion that collagen peptide enhanced BMD in growing rat (Wu, Fujioka, Sugimoto, Mu, & Ishimi, 2004).

Bone marrow is another important part of cancellous bone excluding trabeculae. In newborns, all cancellous bones are filled with hematopoietic marrow, also called red marrow. But as the child ages, the hematopoietic portion decreases in quantity while the fatty portion called marrow adipose tissue (MAT) increases in quantity. Adipocytes, a kind of significant cell in MAT, looks like large bubble in shape, and the number of them is regarded as an indicator of bone marrow adipose degree. Bone marrow fat localizing to the trabecular region, the position of active bone remodeling, indicates its involvement in bone degradation (Lecka‐Czernik, 2012). Emerging evidence is increasingly demonstrating that expanded adipose tissue mass positively correlates with low BMD (Cao, 2011). Adipogenesis is identified to be a default pathway of mesenchymal stem cells (MSCs) which deprive them of the capacity to differentiate into osteoblasts or chondrocytes (Kawai, de Paula, & Rosen, 2012). Proofs have been found that these phenomena existed the same in mice (Duque, 2007; Moerman, Teng, Lipschitz, & Lecka‐Czernik, 2004). We found that our CPs from sharks’ skin can prominently reduce the number of adipocytes, implying that they helped to keep the hematopoietic capability of bone or even restore it, and benefit to the BMD increase, but the CPs from shark's flesh did not possess these effects. The scan electron microscopy detection told us, the abundance of bone marrow has increased due to SCP and SMG after treatment. However, the mechanisms behind the bone‐strengthening effects remain to be clarified. It is significative to figure out which precise peptides contribute to the effects, where they go inside the organism, which signal pathway they participate in and what they become in metabolism.

Heavy metal detection and subacute toxicology assessment helped us estimate the safety of the CPs as functional foods. According to the results, no evidence indicated that SCP and SMG had toxicity to mice at a concentration of 50 mg/ml and a total dosage of 15 g/kg. It is a foundation of their commercial application.

For further industrial outlook of CPs, we are supposed to illuminate the attribute differences between CPs and other protein products such as collagen and collagen peptides. Some researches regard collagen‐rich tissue hydrolysate of aquatic animal as collagen peptides (Huang, Wu, Yang, Li, & Kuo, 2015; Pei et al., 2010; Xu et al., 2010). Such hydrolysates can be broadly called collagen peptides mainly because of their high content of collagen peptides. But technically and strictly, collagen peptides are produced by the hydrolysate of highly pure collagen (Tanaka, Koyama, & Nomura, 2009), making productive process complicated. Thus, we named these collagen‐rich tissue hydrolysate “compound peptides” to identify them. Collagen peptides are more concentrative to some characteristic bioactive oligopeptides, yet CPs possess a wide range of functional components such as essential elements and other bioactive ingredients that collagen peptides do not contain. Hence, there are a few different commercial, healthcare, or medical values between these two products. It is worth mentioning that there is advantage for CPs to compete in the market of nutraceuticals with collagen peptides, because manufacturing process of CPs is more simple and efficient than that of collagen peptides, which is conducive for large‐scale industrial production with low cost.

## 5 CONCLUSION

In present study, SCP and SMG consist of oligopeptides, which are lower than 800 Da, and contain high contents of collagen characteristic amino acids and macroelements as well as safe content of heavy metal elements. SCP and SMG have capability of accelerating bone growth and strengthening bone marrow yet FCP have not. The possible mechanism behind the bone‐strengthening efficacy may be that the SCP and SMG provide sufficient collagen synthetic ingredients to osteoblasts. In subacute toxicology experiment, three kinds of CPs indicated no toxicity to mice. Our research has discovered that SCP and SMG possessed the practical value as functional foods with bone‐strengthening efficacy, which directed an approach for sharks’ skin by‐product utilization.

## CONFLICTS OF INTEREST

There are no conflicts for authors to declare.

## ETHICAL APPROVAL

This research was performed in accordance with the guidelines for the humane treatment of animals set by the Laboratory Animal Center of Xiamen University and the national standards outlined in “Laboratory Animal—Guideline for Ethical Review of Animal Welfare” (GB/T 35892–2018). All animal experiments were approved by Experimental Animal Management and Ethics Committee of Xiamen University (Certificate no. SYXK2013‐0006).
